# Pseudoexpertise: A Conceptual and Theoretical Analysis

**DOI:** 10.3389/fpsyg.2021.732666

**Published:** 2021-11-11

**Authors:** Joffrey Fuhrer, Florian Cova, Nicolas Gauvrit, Sebastian Dieguez

**Affiliations:** ^1^Department of Philosophy, Swiss Center for Affective Sciences, University of Geneva, Geneva, Switzerland; ^2^Department of Psychology, University of Lille, Lille, France; ^3^Laboratory for Cognitive and Neurological Sciences, Unité de Neurologie, Département de Médecine, Université de Fribourg, Fribourg, Switzerland

**Keywords:** expertise, pseudoscience, pseudoexpertise, culture, epistemology, cognition

## Abstract

Some people publicly pretend to be experts while not being ones. They are pseudoexperts, and their presence seems to be ubiquitous in the current cultural landscape. This manuscript explores the nature and mechanisms of pseudoexpertise. We first provide a conceptual analysis of pseudoexperts based on prototypical cases of pseudoexpertise and recent philosophical work on the concept of expertise. This allows us to propose a definition that captures real-world cases of pseudoexpertise, distinguishes it from related but different concepts such as pseudoscience, and highlights what is wrong with pseudoexpertise. Next, based on this conceptual analysis, we propose a framework for further research on pseudoexpertise, built on relevant empirical and theoretical approaches to cultural cognition. We provide exploratory answers to three questions: why is there pseudoexpertise at all; how can pseudoexperts be successful despite not being experts; and what becomes of pseudoexperts in the long run. Together, these conceptual and theoretical approaches to pseudoexpertise draw a preliminary framework from which to approach the very troubling problem posed by persons usurping the capacities and reputations of genuine experts.

## Introduction

During several decades, Stéphane Bourgoin has been considered a prominent expert in the psychology of serial killers and criminal profiling in France. He has been a regular guest on the national French media and is the author of countless books on the topic. According to him, his particular interest in serial killers emerged after the sordid rape and murder of his wife in California in 1976. Although he holds no diploma, he claimed to have benefited from professional training at the FBI quarters. During 30 years, he was, for the media and many professionals, a key expert. Bourgoin also claimed to have been a professional soccer player. However, a recent investigation^1^ revealed that Bourgoin lied on numerous occasions about his past: his wife was not murdered in 1976 (he was not even married), he got no special training from the FBI, and he did not meet the 77 serial killers he claimed to have personally interviewed. His career as a footballer is also a fabrication. Bourgoin eventually confessed to his lies. Can he still be considered an expert on the topic of forensics and profiling? Or was he more like a pseudo-expert all along?

If experts could be reliably recognized as such, there would be no discussion about a current “crisis of expertise.” Yet, some have noted a worrying trend in our current intellectual environment: actual experts are sometimes ignored and dismissed, while non-experts tend to pose as and take the place of genuine experts (Nichols, 2017; Eyal, 2019). This leads to two questions that we address in this manuscript: (i) what is pseudoexpertise and what are its core features?; (ii) how can some pseudoexperts be so successful and what makes the content they produce so attractive to laypeople?

To approach these questions, we will use two different methodologies. For the first one, we will focus on conceptual analysis to identify what pseudoexperts and their core features are, basing our reflections on recent philosophical work on the question of expertise (section “What Are Pseudoexperts”). This will provide the ground for our second question to build a general and theoretical framework, using the cognitive approach of cultural attraction theory (CAT), also known as cultural epidemiology (section “A Framework for Further Research: Three Questions About Pseudoexpertise”). This second aim will be presented in the form of three issues to be addressed by further research on pseudoexpertise, namely why it exists at all, how it can be successful despite its obvious shortcomings, and how it evolves dynamically.

## What Are Pseudoexperts?

If we are to investigate pseudoexperts and the reasons for their success, we first need to delineate the phenomenon of pseudoexperts to determine what exactly needs to be studied and where to draw the line between pseudoexperts and other related but different constructs. In this section, we begin by surveying some of the ways in which the expression “pseudoexpert” is used and then propose a characterization of pseudoexperts that tries to do justice to most of these usages, while capturing the main concerns about pseudoexperts. Indeed, if there is at all a concept of pseudoexpertise that points to real-world phenomena, an adequate definition of pseudoexperts should fulfill three conditions: (i) it should reject most obvious instances of non-pseudoexperts (and most notably genuine experts); (ii) it should include most obvious instances of pseudoexperts; (iii) it should capture and justify our main qualms with the existence of pseudoexperts. This is where we start our investigation of pseudoexpertise.

### Some Uses of the Expression “Pseudo-Expert”

Previous uses of the expression “pseudo-expert” offer rather diverse and contrasting views. For instance, in 1897, the medical doctor and lawyer Henry Smith Williams worried about the increasing presence of “pseudo-experts” in criminal trials acting as expert witnesses, and demanded that “the law should at least make the effort to discriminate between the real and the pseudo-expert” (Williams, 1897, p. 161). In this author’s view, having “average (.) knowledge” or a “good professional standing” (p. 162) wasn’t enough. He asked for an official process of certification based on education and specialization, checks for moral standing, and a specific examination assessing mastery of the topic at hand, and he opined that “common sense” would also require some track record and experience in the domain: “These restrictions alone would bar out from eligibility for examination even a large proportion of the pseudo-experts who have made expert testimony a jest and a reproach” (p. 163). A later observer more specifically deplored that pseudoexperts in the courtroom are witnesses uninformed of the current state of their field, and also “incapable of presenting [the latest data] properly” (Smith, 1930, p. 90). That is, beyond being merely incompetent or unqualified, pseudoexperts would in addition be *asked to perform as* experts, leading them to communicate an erroneous image of the state-of-the-art of a specific domain to the public. Pseudoexperts, thus, have a bad knowledge of a field and communicate poorly to the public about that field, and yet they are presented and present themselves as experts of that field.

More recently, Sorial (2017) investigated the role of “pseudo-expertise discourse” in “denialism” (specifically, the attempt at giving the appearance of a legitimate scientific debate where none exists). She focuses on the rhetorical uses of mimicking strategies by pseudoexperts, that is, how they attempt to *pass* as experts. Her “phoney experts” are “speakers who attempt to use the tropes of expertise to manufacture legitimacy for their views, such that they *appear* to have technical knowledge even though they actually do not” (p. 305, emphasis in original; Sorial also talks of “false scientific speech,” p. 322). Thus, in this view, pseudoexpertise is a cynical attempt at usurping the status afforded by expertise for further ends (of a propagandist, ideological, or political nature). Of note, Sorial allows the possibility for actual experts to be also pseudoexperts. She takes the example of Peter Duesberg, an actual scientist who notoriously uses his status to defend a denialist view of AIDS.

Lastly, Goldman (2018) uses the term “pseudo-expert” to refer to people who claim a position of expertise to justify ideological and nefarious agendas, presenting as “fact” what is in reality ideology. In this sense, accusations of pseudoexpertise have a decidedly moral overtone: pseudoexperts are not only persons who “don’t genuinely possess any greater body of truths in the relevant domain that they claim to have,” but also persons who exploit the pretense of expertise to help enforce unethical policies, or push their own political agenda.

These examples show the multiple meanings of pseudoexpertise, which involve personal, social, moral and ideological aspects. It can denote lack of qualifications, lack of knowledge, failure to keep one’s knowledge up-to-date, failure to accurately communicate the consensus knowledge of the field, and deliberate exploitation of the prestige and credibility afforded to expertise for nefarious aims.

### From a Characterization of Experts to a Definition of Pseudoexperts

In this section, our goal will be to develop a definition of pseudoexperts that will account for these different cases, while explaining what’s wrong with pseudoexpertise. As a first step to an adequate definition of pseudoexperts, we will start from a characterization of genuine experts, as pseudoexperts will necessarily be defined in relation to them.

There is no universally accepted definition of expertise and experts (Quast and Seidel, 2018). However, our goal here is not to provide yet another definition of experts and expertise and defend it against other views. Rather, we will draw on the already existing definitions and accounts of expertise that have been deployed by philosophers to highlight some characteristic features of experts and to distinguish between different meanings of expertise.

A traditional approach to expertise highlights the superior knowledge and skills (or “know-how”) of experts. On this *ability-based* approach to expertise, experts possess objective and often measurable skills and cognitive abilities that distinguish them from non-experts (Simon and Chase, 1973). In the context of scientific expertise, one obvious way of distinguishing experts from non-experts is to define people who have more knowledge (or more true beliefs) than others on a certain topic. Thus, Goldman (1999) defines the authority of experts in the following way:

Person A is an authority in subject S if and only if A knows more propositions in S, or has a higher degree of knowledge of propositions in S, than almost anybody else (Goldman, 1999, p. 268).

However, some have argued that such definitions in terms of knowledge or true beliefs are insufficient. Croce (2019) asks us to imagine the case of someone who learns most of what is known in a given domain by heart, without really understanding any of it, and argues that such a person, despite their superior knowledge, and would not count as an expert. Thus, it is often considered that the expert’s greater knowledge must be accompanied by a certain number of abilities. Scholz (2018) and Croce (2019) argue that the true expert must not only know the truth of a certain number of statements about their topic of expertise, but that they also need to *understand* them, where understanding is defined as “grasping systematic connections among elements of a complex whole, or gaining insight into certain relations between items within a larger body of information” (Jäger, 2016, p. 180). In addition to knowledge, Goldman (2001) emphasizes the two following abilities as characteristic of genuine experts:

• The *ability to form new reliable beliefs*. When asked a question about their field of expertise they do not know the answer to, genuine experts know *how* and *where* to search for the relevant information. This might involve knowing both knowing where to find a reliable answer to the question when there is already one, or what kinds of operation to perform to produce new reliable information on the topic.
• A *second-order knowledge of their field of expertise*. Experts don’t only have knowledge *in* their fields, they also have knowledge *of* their field. That is, they know which questions are considered as settled by other experts in the field and which ones are still considered as open and controversial.

These characterizations of experts and expertise focus on dispositions (knowledge and skills) that are *internal* to experts, in the sense that they define or characterize expertise without reference to the experts’ environment and the context in which they manifest their expertise. Other approaches to expertise can be considered as *external*, as they consider that experts have to be defined in relationship with the social environment they operate in. A radical brand of external characterization is to consider that someone is an expert if and only if they are *considered* as such. To put it bluntly, to be an expert and to be considered as an expert are, in this view, one and the same thing. Putting aside its radicality, it is clear that such a definition won’t help us understand what is the problem with pseudoexperts. Indeed, if being an expert is the same as being considered as an expert (or having a reputation for expertise), and if a pseudoexpert is someone who passes as an expert without being one, then this definition of expertise will lead us to conclude that a pseudoexpert is someone who claims to be widely considered as an expert while almost no one actually consider them to be an expert. This would mean that what differentiates the pseudoexpert from the genuine expert is only the number of supporters they can boast. But if that is the only difference between experts and pseudoexperts, then we cannot understand why we should worry that people listen to pseudoexperts rather than experts (see section “The Problem With Pseudoexperts”).

A more useful external approach to expertise is the one that considers that experts are to be defined by the *social role* they play. Under this approach, expertise is not defined only by the possession of certain abilities, but also by the function experts are supposed to play in the division of epistemic labor. According to such accounts of what it is to be an expert, speaking of “expertise” requires one to make some (minimal assumptions) about the epistemic organization of one’s society. Indeed, speaking of experts suggests that there are topics about which not all people have equal knowledge, and it also suggests that certain people play a specific role that requires them to have and foster superior knowledge about this topic. To put it otherwise, the term “experts” presuppose some form of social division of epistemic labor, in which certain persons play a specific epistemic role (Sterelny, 2012).

But what is exactly the role of experts? Croce (2019) distinguishes two different functions of experts (even if he only takes the first one as being the *true* function of experts). On a *research-oriented* account of experts, the function of experts is to contribute to the epistemic progress of the field they are experts in. In this view, the role of experts is to contribute to the expansion of knowledge in their field, mainly by conducting new research. However, on a *novice-oriented* account of expertise, the function of experts is to be “someone laypeople can go to in order to receive accurate answers to their questions” (Coady, 2012, p. 30). On this view, the role of experts is to discharge non-experts from the need to become experts themselves to find the information they need about a given topic. Of course, the same person can fulfill both functions (and one could even argue that it is best if someone who fulfills the *novice-oriented* function also fulfills the *research-oriented* one). However, these two functions can sometimes come apart. We could imagine a recluse scientist who prefers to focus on their research rather than interact with laypeople, as we can imagine someone who keeps up with the scientific research and transmits its result to non-experts without contributing (much) to the development of their field.

We do not think that one of these two accounts of the function of experts is better than the other, rather they capture two distinct roles experts can play (often simultaneously) within a given society^2^. However, in this manuscript, we will focus on experts defined as those who play the *novice-oriented* function of providing non-experts with information and help that only experts have access to. The reason is that this definition seems more suited to capture the kind of cases we usually apply the qualifier “pseudoexperts” to (as overviewed in section “Some Uses of the Expression “Pseudo-Expert””). Indeed, supposing that we define the pseudoexpert as someone who poses as an expert in the *research-oriented* but not in the *novice-oriented* sense, what would such a pseudoexpert look like? They would claim that they make substantial contributions to their field of expertise, but would refuse to share their “knowledge” with non-experts (or other experts). This would be in stark contrast with paradigmatic cases of pseudoexperts, who seem very happy to share their wisdom with non-experts, which makes them all the more dangerous.

As such, we might define experts as those whose function involves being ready and able to help people who are, or apparently are, less knowledgeable and skilled find the information they need and would not be able to find by themselves. Thus, Goldman (2018) defines what it takes to be an expert in the following way:

S is an expert in domain D if and only if S has the capacity to help others (especially laypersons) solve a variety of problems in D or execute an assortment of tasks in D which the latter would not be able to solve or execute on their own. S can provide such help by imparting to the layperson (or other client) his/her distinctive knowledge or skills. (Goldman, 2018, p. 4)

Defining experts by their function (or role) presents an additional advantage: it stresses the fact that experts have *duties* toward the people they are supposed to help, and thus must comply with certain *norms* (Sorial, 2017). Indeed, it is always possible for something that has a function to malfunction, i.e., to fail to fulfill this function. To fulfill their function, experts thus have to comply with certain norms inherent to this function: for example, they must be truthful, be mindful of presenting the consensus rather than their own opinion, and strive to keep up with the state-of-the-art in their area of expertise (Hardwig, 1994).

However, what constitutes the *capacity* to fulfill a certain role is not easy to delineate. In a narrow sense, this capacity includes only the knowledge and skills already highlighted by the ability-based approaches to expertise. But *capacity* can also mean benefiting from the social circumstances that would allow one to exercise one’s knowledge and skills. Thus, Quast (2018) argues that someone who has the required knowledge and skills but is isolated from other humans, such as Robinson Crusoe, would not count as an expert, since “there is nobody for whom he can function as an expert or even no nearby counterfactual scenario in which he could plausibly do so” (p. 25). Pushing this line of reasoning further, some even argue that being an expert requires being recognized as such (even though being recognized as an expert is not sufficient to be one). Indeed, to fulfill their function, experts need to be listened to and trusted, and thus must be recognized as being competent in their area. To act as experts, they must benefit from a certain *status* and enjoy some *epistemic authority*. This is why “being credited with expert status can confer power and authority” (Stichter, 2015, p. 125) and why Quast (2018) claims that there is an evaluative sense of the word “expert” in which “expertise represents an honorific term, for experts by definition are set to accurately fulfill difficult service-functions (.) and are thought not only to be competent enough to do so, but also to creditably and responsibly manifest pertinent competences thereby” (p. 25).

But if an expert is someone who is able to fulfill a certain function, and if fulfilling this function requires one to be granted some status, why not conclude that it is necessary to be granted this status to be an expert in the full sense of the term? This is the conclusion some have argued for. For example, Agnew et al. (1997) claim that, to be an expert, one needs “to have at least one reasonably large group of people (…) who consider that you are an expert; in this sense, expertise is socially selected.” Likewise, Stichter (2015) argues that “we confer the status of experts on others (along with whatever power goes along with it)” and that we should “avoid talking in terms of “recognizing” or “identifying” experts, as that may suggest people are experts independent of us conferring that status on them” (p. 126).

Here, we do not need to defend such a narrow view of experts. Rather, what we want to highlight is that, to fulfill their *novice-oriented* function, experts must be recognized as such by their audience and, thus benefit from a certain epistemic authority and of certain advantages and power that comes with it.

To sum up, while surveying accounts of experts and expertise, we highlighted three key conditions for expertise. In the fullest sense of the term, an expert (in the *novice-oriented* sense) is someone who (i) possesses expertise, such as that they possess superior knowledge and skills in a given domain *d*; (ii) uses this expertise to fulfill a certain function inside a given epistemic community – i.e., helping *novices* (i.e., non-experts) access knowledge about *d* that is normally only accessible to experts; and (iii) benefits from a certain social status and epistemic authority, on the basis of public recognition of their having the ability and willingness to fulfill this function.

Note that we do not present this definition of expertise as the *right* one or the only possible one (as we mentioned earlier, a *research-oriented* definition of expertise might be equally suitable for other goals). Rather, we take this definition as the one required to highlight and define the phenomenon of pseudoexpertise. Indeed, taking stock of this definition of expertise, we propose to define pseudoexpertise in the following way:

A pseudoexpert is someone who (C1) seeks to be granted by non-experts the social status typically granted to experts in domain *d* in the *novice-oriented* sense; and (C2) engages in behaviors related to *novice-oriented* function of experts in domain *d*; (C3) while being either *unable* to fulfill the related *novice-oriented* function (because they don’t have the required degree of knowledge and skill in the domain) or *unwilling* to fulfill it (because they don’t want to comply with the norms inherent to this role); (C4) while there are people with expertise in domain *d* who fulfill or would fulfill this function in a better way.

The idea behind this definition is the following: because being able and willing to fulfill the *novice-oriented* function or expertise often comes with the enviable social status of expert, the pseudoexpert is the one that seeks to be granted this status by non-experts to reap the associated benefits without actually being able or willing to properly fulfill the related function. These benefits can be either personal or ideological.

### Distinguishing Pseudoexperts From Other Types of Non-experts

As we said earlier, one criterion for an adequate definition of pseudoexperts is that it allows us to distinguish pseudoexperts from other related but different constructs. Here, we argue that our definition succeeds in differentiating pseudoexperts from other brands of non-experts.

First, our definition allows us to distinguish pseudoexperts from *epistemic trespassers*. Ballantyne (2019) defines epistemic trespassers as “thinkers who have competence or expertise to make good judgments in one field, but move to another field where they lack competence—and pass judgment nevertheless” (p. 367). His examples include evolutionary psychologist Richard Dawkins writing about the psychological underpinnings of religious belief, astrophysicist Neil deGrasse Tyson giving his opinion about philosophical matters (Ballantyne, 2019), the Nobel double-laureate Linus Pauling promoting vitamin C against cancer, or sociologists supporting intelligent design (such as Fuller, 2008). Though most pseudoexperts typically will engage in epistemic trespassing, one can engage in epistemic trespassing without being a pseudoexpert. Indeed, when Neil deGrasse Tyson makes a remark about philosophy, he does not claim to be an expert in this field, nor to be recognized as an expert in the field of philosophy. Thus, epistemic trespassers do not necessarily fulfill condition (C1).

Second, our definition distinguishes pseudoexperts from *pundits* and *science journalists and popularizers*. Pundits (or *editorialists*) are people who regularly intervene in the media to express themselves on a wide array of topics, so that they cannot be plausibly experts in all these fields. They typically display high levels of overconfidence and heated rhetoric, so that they may sound as if they must know what they are talking about. However, we should not count them as pseudoexperts, since they usually don’t claim to be experts, or seek to be recognized as such, and thus do not satisfy condition (C1). Rather, pundits will tend to claim or imply that they are educated non-experts, who draw on a wide range of general knowledge, common sense and intuition to ‘‘transcend’’ the ‘‘short-sightedness’’ that comes with true expertise. Science journalists and popularizers, on the other hand, typically claim to be knowledgeable about (some areas of) science. As such, they claim to have some degree of expertise (in a *novice-oriented* sense)^3^. However, they typically do not claim to have *as much* expertise as scientific researchers, or to usurp their status, and so usually refrain from engaging in (C1). Indeed, they typically resort to genuine experts instead of trying to pass as one, and therefore usually do not resort to (C2) either.

Third, our definition allows us to distinguish pseudoexperts from *crooks* and other *con-artists* whose goal might be to obtain money or other favors from their preys. The difference here is that conmen are not primarily interested in passing as experts, and will only do so when it serves their immediate purposes. For example, it will be enough for them to convince their prey that they are recognized as experts, even if they are not. Thus, *crooks* and *con-artists* do not necessarily satisfy condition (C1), as they do not seek to be granted the status of experts by society, but can be content with merely pretending that they have been granted such a status. In fact, they do not even need to fulfill (C2) at any time: they would be content to merely pass locally and temporarily as someone would *could* engage in (C2).

Fourth, defining pseudoexperts as people who claim to fulfill the *novice-oriented* function of expertise, rather than the *research-oriented* one allows us to exclude cases of *bad researchers*. A researcher can be bad in two different ways. On the one hand, researchers can be bad because they do not want to comply with norms researchers are supposed to follow. For example, social psychologist Diederik Stapel is known for having published falsified data and even created “data” *ex nihilo* (Callaway, 2011). Note that he has been called many things, among which a *fraudster*, but not a pseudoexpert. On the other hand, researchers can be bad because they lack the skills and competence to contribute to the progress of their field. We can imagine a researcher who, through a series of unfortunate events, obtained his Ph.D. then got hired by a university even though their research skills are lacking. In both cases, to the extent that these researchers do not try to act as a public experts but only limit their activities to research, they will not fulfill (C2) and won’t be counted by our definition as pseudoexperts. This allows our definition to capture the fact that the label “pseudoexpert” is mostly used to refer to persons presented and posing as “experts” in the public sphere, sharing information with non-experts, and to distinguish between pseudoexperts and “pseudoresearchers.” For the same reason, focusing on the *novice-oriented* account of expertise rather than the *research-oriented* one also allows us to differentiate pseudoexperts from *crackpots*, who are (falsely) convinced to contribute to the progress of a given domain of research. These people are actively seeking to share their discoveries with genuine scientists, but are not necessarily interested in sharing their knowledge with the general audience at large [thus failing to fulfill the *novice-oriented* component of (C2)].

Finally, through condition (C4), our definition distinguishes pseudoexperts from pseudoscientists. Indeed, (C4) captures the idea that pseudoexperts are people who compete with genuine experts to usurp their status (hence the *pseudo-* in “pseudoexperts”). Thus, according to our definition, there can only be pseudoexperts in fields in which *there are* experts. However, since expertise requires a considerable amount of knowledge, this means that there can only be pseudoexperts in fields where there is knowledge to be found in the first place. But pseudosciences are typically fields in which no knowledge, and thus no expertise, is possible. There is no real expertise in spirit invocation or crystal healing, and thus, by (C4), there are no pseudoexperts in such matters either, only pseudoscientists or pseudo-therapeutes. One might object that pseudoscientists often make assertions that run directly contrary to established science, and that this makes them direct competitors of genuine scientific experts. However, being in competition with genuine scientific experts does not mean that one claims to be an expert in the field of these scientific experts. For example, a creationist priest who claims that humans were created by God and did not evolve from previous different species is in competition with genuine scientific experts. However, this priest will not be a pseudoexpert as long as he does not claim to be an expert in evolutionary biology and strive to pass as one. Thus, not all competitors to genuine scientific experts are pseudoexperts.

Another objection might simply be that some pseudoscientists explicitly claim to be *experts* in their field and, as such, are mimicking expertise *at large*. Thus, we might be tempted to call them “pseudoexperts” after all, and that would be one point where our definition possibly does not fully match the everyday use of the term. Nevertheless, we think such cases would be better distinguished from cases of pseudoexperts as we defined them, as the challenges that pseudoexperts and pseudoscientists face are quite different: pseudoscientists have to argue for the legitimacy of their field while pseudoexperts benefit from the established legitimacy or the field they infiltrate. Of course, there are complicated cases: for instance, a physician promoting homeopathy and doing so on the grounds of his expertise in medical sciences could count both as a pseudoscientist and as a pseudoexpert. In fact, pseudoscientists will often be pseudoexperts in the fields directly adjacent to their specific brand of pseudoscience, at least to the extent they attempt to pose as experts in those fields in order to bolster their pseudoscientific claims: a crystal healer will tend be a pseudoexpert in both mineralogy and medicine, an astrologer would tend to be a pseudoexpert in astronomy and psychology, a conspiracist will tend to be a pseudoexpert in geopolitical matters and history, and so forth.

### Capturing Typical Cases of Pseudoexperts

A second desideratum for our definition of pseudoexperts was that it should accommodate most (if not all) prototypical instances of pseudoexperts. As we will see, it succeeds in capturing the instances we collected in section “Some Uses of the Expression “Pseudo-Expert.”

Some of these instances highlighted the fact that pseudoexperts are characterized by a lack of skill and knowledge. These cases of pseudoexperts are captured by our definition, when it identifies as pseudoexperts people who seek to be recognized as experts while lacking the skills necessary to fulfill the related function. This includes the case of Stéphane Bourgoin, which we described in the introduction. Stéphane Bourgoin presented himself as an expert in the psychology of serial killers and criminal profiling and has reaped the benefits that come with this status (for example, by selling many books purporting to introduce non-experts to criminology). What is more, Bourgoin let himself be presented as a world-renowned expert by many journalists. As such, Bourgoin fulfills conditions (C1) and (C2). Since there are genuine experts in his field, condition (C4) is also fulfilled. However, as we saw, Bourgoin also lacked the training and thus the skills required to properly fulfill the expert’s *novice-oriented* function which means that he also fulfills condition (C3). Thus, on our definition, Bourgoin counts as a pseudoexpert.

Another case in the same range would be Jean-Dominique Michel, whom some have described as a “self-proclaimed expert” (Garcia, 2020). Jean-Dominique Michel has presented himself as a medical anthropologist and a public health expert, which allowed him to be invited several times by numerous Swiss media to discuss matters relevant to the COVID-19 pandemic. Thus, Jean-Dominique Michel fulfills conditions (C1) and (C2) and, since there are genuine experts in the fields of medical anthropology and public health, he also fulfills (C4). However, as it turns out, Jean-Dominique Michel seems to lack training in the areas he claims to be an expert in: he has neither a Ph.D. nor even a Master’s degree in the field of anthropology. If we take this as indicating the lack of competence in this domain, then the case of Jean-Dominique Michel also fulfills condition (C3), which makes him a pseudoexpert on our definition.

However, not all instances of pseudoexperts we surveyed involved a lack of competence. Others were more focused on the morality of expertise and counted as pseudoexperts people who did not necessarily lack competence, but used their expert status for political, ideological and even selfish ends. Such cases are captured by the part of (C3) that counts as pseudoexperts people who refuse to comply with the norms inherent to the role experts are supposed to play. Indeed, a simple but fundamental norm of *novice-based* expertise is that experts should strive to provide reliable information to the ones seeking their advice. This involves painting a fair picture of the scientific consensus (or lack of consensus) on the topic, rather than promoting one’s own personal opinions. Thus, someone with true expertise (in the sense of having the required knowledge and skills, as well as credentials) can still be a pseudoexpert in this sense. One example would be French microbiologist Didier Raoult, who was the first to promote hydroxychloroquine as a treatment against COVID-19. Though this hypothesis might have been reasonable at the time, when appropriate data was lacking, there is now ample evidence that this treatment does not work. Still, in spite of the evidence, Didier Raoult continues to promote this treatment and has taken the habit of bypassing the scientific community to directly address the public through media and his research institutes’ own YouTube channel (which, at the moment we write this manuscript, can boast a total of 500,000 followers) (for a summary of this debate in the French context, see Fuhrer and Cova, 2020; Schultz and Ward, in press). Thus, it can be said that (C1) Didier Raoult seeks (and has actually achieved) the status of scientific experts in the eyes of non-experts, (C2) that he engages in activities related to the *novice-oriented* function of experts, but (C3) that he seems more interested in communicating his ideas rather than presenting his audience with a fair picture of the scientific consensus. As (C4) there are experts more suited to this task in his field, that would make Didier Raoult a pseudoexpert on our definition – though for very different reasons compared to Stéphane Bourgoin and Jean-Dominique Michel.

Thus, our definition seems to capture prototypical cases of pseudoexperts while providing a distinction between two types of pseudoexperts: *incompetent* and *malevolent* pseudoexperts^4^.

### The Problem With Pseudoexperts

One final desideratum for our definition of pseudoexperts was that it should explain why pseudoexperts are a problem, and even a danger. We think our definition fulfills this requirement in a quite straightforward way: according to our definition, pseudoexperts seek to trick people into thinking that they are both able and willing to provide them with reliable information on a topic, while being unable and/or unwilling to do so. As such, pseudoexperts participate to the spread of misinformation and can, in the long run, undermine non-experts’ trust in experts (either because they contradict genuine experts, or because they are ultimately exposed as fraud, shedding doubt on the reliability of other people also recognized as experts). Pseudoexperts thus represent a threat not only to the lay audience that might be misled by their claims, but to the concept of expertise itself, in that pseudoexperts undermine the necessary trust involved in relying on expert claims which, by definition, non-experts are in no position to assess objectively. As such, pseudoexpertise, like other forms of disinformation and misinformation, is a threat to the very foundations of knowledge in liberal societies^5^.

## A Framework for Further Research: Three Questions About Pseudoexpertise

Now that we have identified pseudoexpertise as a distinct phenomenon, we would like to explore some specific questions posed by its existence, and suggest potential lines of research for the future.

### What Makes Pseudoexpertise Possible?

The first question raised by the existence of pseudoexperts is simply: how are pseudoexperts possible? This might seem a very naive question, but we should keep in mind that people are not as gullible as some might think. Indeed, people tend to distrust unreliable and incompetent sources (Heyman, 2008; Sperber et al., 2010; Harris and Corriveau, 2011), and are usually quite able to distinguish between false and true information (Pennycook and Rand, 2019; Altay et al., 2020b; Bago et al., 2020; Berriche and Altay, 2020; Mercier, 2020). So, why would they trust pseudoexperts that will typically be unreliable?

One answer to this question might be found in the fact that expertise is a largely *opaque* property of epistemic agents, leading to troubles in expert identification. This is nothing new: in *Charmides*, Plato already claimed that only an actual medical doctor will be in a position to unmask a fake doctor. Indeed, to truthfully identify an expert, one would need to become one, which seems absurd, considering that the point of expertise is to allow for the division of epistemic labor (Sterelny, 2012). Novices and non-experts need experts precisely to the extent that they are not and (for the most part) will never be experts themselves. However, because they are not experts, they are in no position to discern exactly what it takes to be one, and exactly why experts are experts.

Nevertheless, one might think that Plato’s conclusion is too pessimistic. Aristotle did, and argued that non-experts can sometimes evaluate the output of experts: laypeople can estimate the work of a shoemaker by wearing the shoe and feel whether it is comfortable or not. Thus, people are not completely blind when it comes to evaluating experts. Goldman (2001) lists four type of cues laypeople can use to assess experts:

(A) Arguments presented by the contending experts to support their own views and critique their rivals’ views.
(B) Agreement from additional putative experts on one side or other of the subject in question.
(C) Appraisals by “meta-experts” of the experts’ expertise (including appraisals reflected in formal credentials earned by the experts).
(D) Evidence of the experts’ interests and biases vis-a-vis the question at issue.
(E) Evidence of the experts’ past “track-records.”

Thus, we can expect pseudoexperts to flourish in domains in which these different cues become difficult to assess.

Let’s take the example of (E) experts’ past track records. This is precisely what Aristotle suggested with his example of the shoemaker: laypeople can judge experts based on their outputs. In the intellectual and scientific domain, one such output might be predictions. This is why, when Tetlock (2005) set out to assess political expertise, he focused on political experts’ ability to make precise predictions (forecasting). But one of Tetlock’s main points was that such an assessment could hardly be made by laypeople because experts’ predictions in the political domain are typically probabilistic or ambiguous – or, even worse, ambiguous about probabilities. Indeed, it is hard to assess the truth of predictions such as “the debt crisis in Europe (.) may be very close to going critical” (Tetlock and Gardner, 2016). How probable is “may” and what does “critical” mean?

In other domains, outputs can be non-ambiguous, but their assessment can be out of reach for non-experts. For example, an expert mathematician’s output will be precise and non-ambiguous, but it will consist of demonstrations and theorems that will be arcane to laypeople. Thus, we can expect that pseudoexperts will be more likely to flourish in areas in which outputs are either too vague or inscrutable for non-experts.

As such, it seems that the phenomenon of pseudoexpertise is one of exploitation of the very opacity of genuine expertise to non-experts. A pseudoexpert might choose to mimic any of Goldman’s criteria listed above in order to pass as an expert.

### What Strategies Make Pseudoexperts Successful?

As we defined them, pseudoexperts are in competition with experts. That is, pseudoexperts operate in epistemic landscapes where we can find agents who are reliable and eager to provide scientifically accurate information. Thus, pseudoexperts can be considered as free-riders searching to take advantage of some of their epistemic landscape’s cracks and loopholes in such an epistemic landscape. One valuable topic of investigation might be to identify the strategies that allow pseudoexperts to succeed in these epistemic landscapes. This topic of investigation can be broken down in two sub-questions that correspond to two different challenges pseudoexperts have to meet:

• The *Legitimation* Challenge. Because pseudoexperts want to be considered as experts, they must convince people that they possess the required skills and knowledge – i.e., that they are *legitimate* experts.
• The *Attractiveness* Challenge. Because the pseudoexperts’ goal is to reap the benefits that come with being recognized and consulted as experts (including the mere fact of passing as an expert), they also need to be selected over other genuine experts and candidate pseudoexperts. As such, their pronouncements need to be more or at least equally *attractive* than those of others to their audience.

How do pseudoexperts meet these challenges? In this sub-section, we would like to offer some tentative answers to these questions, which might serve as hypotheses for further research. However, though they are grounded in personal observations and existing scientific frameworks, the considerations that follow must be taken as merely exploratory^6^.

#### Meeting the Legitimation Challenge: Diplomas and Other Signs of Institutional Recognition

As we just mentioned, a first step in becoming a successful pseudoexpert is to convince one’s audience that one has enough skills and knowledge in a given area. To this end, pseudoexperts can emphasize their diplomas and affiliation to certain institutions. Indeed, those are social markers of expertise and reputation (and therefore competence) that are theoretically accessible to non-experts, though laypeople will not always have a perfect understanding of how institutional recognition works. Regarding diploma and affiliation to research institutions, the best case is when the pseudoexpert indeed received a diploma in a relevant or adjacent field and is a member of a research institution. In other cases, the pseudoexpert will have some diploma, but not in the relevant area of expertise. In case they lack the relevant titles and diploma, pseudoexperts might attempt to stretch their titles to give the (false) impression that they are somehow relevant. For example, French essayist and coach Idriss Aberkane claimed to have a Ph.D. in neurosciences (and thus to be an expert in this field), while his Ph.D. was actually a Ph.D. in management (Durand, 2016). Finally, in the worst-case scenario, pseudoexperts might lack the relevant titles. In this case, it is always possible to lie about one’s titles or to find shady organisms that might be complacent enough to deliver what looks like a diploma.

The same goes with institutions. Pseudoexperts will boast about their affiliation to institutions that laypeople might perceive as prestigious (e.g., Stanford and Harvard). And a pseudoexpert who lacks affiliation to legitimate research institutions might try to exaggerate certain interactions with legitimate institutions – for example, pretending to have been an invited researcher in a certain university just because one was once invited to give a conference by a student association. Another possibility is to follow the methods of pseudoscience and create one’s own parallel institutions, the name of which mimics the name of legitimate institutions.

Such signs of institutional recognition will probably be of special importance to many pseudoexperts because, most of the time, their work won’t be recognized (and will often be actively dismissed) by genuine experts. However, diplomas are interpreted by laypeople as a form of recognition from the academic community. As such, we can expect that one difference between experts and pseudoexperts is that the first will be more likely to ground their expertise on their current work, while the latter will be more likely to refer to their diplomas and academic titles.

Finally, let’s note that signs of institutional recognition might also be used by pseudoexperts to meet the attractiveness challenge and differentiate themselves from genuine experts. For example, Idriss Aberkane styles himself as a “hyper doctor” because he can boast three Ph.D’s (obtained in 3 years) in three different domains.

#### Meeting the Legitimation Challenge: Outputs and Track-Records

As highlighted by Goldman (2001), experts’ outputs and track-records are signs people use to assess experts’ skills and competence. However, what counts as a legitimate scientific output is again partly opaque to non-experts. For example, it is not clear whether most non-experts, including journalists, understand the difference in value between a scientific manuscript published in a peer-reviewed journal and a book published by a non-academic press. This gives pseudoexperts some leeway to exaggerate their outputs and track-records. When they fail to produce scientifically legitimate content (such as scientific manuscripts), pseudoexperts might resort to several alternative strategies, such as non-scientific publishing, preprints, or predatory journals. Here again, pseudoexperts might also draw on their outputs and track record to make themselves more attractive.

Since the quality of scientific productions is mostly opaque to non-experts, we can also expect pseudoexperts who have this possibility to emphasize the volume or quantity of their contribution, and thus to abuse metrics such as number of citations or number of books and manuscripts (as is the case with Didier Raoult’s voluminous and fast-paced publication record mostly in friendly or in-house publication outlets).

#### Meeting the Attractiveness Challenge: The Content of Pseudoexperts’ Discourse

Experts in the media are often asked to explain their claims and predictions, and to defend them against competings claims by other experts. Goldman (2001) sees that as an opportunity for laypeople to assess the experts’ skills by evaluating the soundness of their argument. But Goldman is also aware that such cues can potentially be abused, and that non-experts might be sensitive to cues that are only indirectly related to expertise, such as rhetorical style. More generally, to the extent that experts’ discourse might be partially opaque to non-experts, pseudoexperts might seek to score points against genuine experts by making their content more *attractive*. Indeed, because pseudoexperts are not bound by their willingness to be accurate, they have more leeway to tailor their discourse to please and capture their audience (such an insensitivity to truth is the hallmark of what Frankfurt, 2009 calls “bullshit”). Here, we draw on CAT to highlight several ways in which pseudoexperts can tailor their discourse.

(1) In addition to accuracy and internal coherence, one of the foremost cues in human communication is an expectation of *relevance* (Sperber and Wilson, 1995). *Relevance* can first be construed in a cognitive sense, in which information is relevant when it is novel, surprising and attention-catching. Thus, Altay et al. (2020a) found that some misinformation is shared not because people think it true, but because if it were, it would be interesting. Moreover, slightly counterintuitive claims are more easily memorized (Boyer and Ramble, 2001; Boyer, 2003; Banerjee et al., 2013). One risk of making counter-intuitive claims might be that such claims are more easily falsified, but some of these claims can be made ambiguous enough to be surprising on one reading and trivial on another (this is what Dennett, 2009^7^ calls “deepities”). We can then expect pseudoexperts to abuse surprising claims and anecdotes.
  However, relevance can also be construed in a motivational sense, in which information is relevant when it bears some connection to our interests and well-being. Thus, evidence again indicates that people’s standard for evidence lowers when a claim suggests the existence of a threat (Fessler et al., 2014; Nera et al., 2021). We can then expect pseudoexperts to focus on claims relevant to their audience’s well-being, with a tendency to highlight potential threats.
(2) Another strategy might be telling people what they want to hear – i.e., presenting them with information congruent with their values and worldviews. Indeed, we tend to prefer evidence that confirms our pre-existing beliefs (“myside bias,” see Mercier, 2016). Thus, to distinguish themselves from genuine experts, pseudoexperts might choose to systematically present accounts their public would wish were true. One way to do that is to use what Dieguez (2018) calls the “inverse guru effect”: presenting ideas that are common and likely to be already shared by the pseudoexperts’ audience, but in a form that makes them sound “deeper” and more “profound” than they really are, especially when coming from a seeming expert.
(3) Pseudoexperts can also favor claims and arguments that tap into people’s *intuitions*. Indeed, the psychological literature has stressed that certain concepts are more intuitive than others, because they fall in line with automatic cognitive processes and biases. For example, they can share contents that resonate with our intuitive theories, such as essentialist theories that capitalize on folk biology (Gelman and Gottfried, 1993), or intentional explanations that will please our folk psychology (Dennett and Haugeland, 1987; Dennett, 2009). Such content bias has been shown to have a major role in cultural transmission (Berl et al., 2021) and is supposed to also play a role in the success of various pseudosciences (Boudry et al., 2015). Tapping into these intuitive processes might allow pseudoexperts to offer cognitively attractive narratives.
(4) Finally, pseudoexperts might *oversimplify* the content of their discourse. The methods and results of science are not always easy to convey, and the expert might not always be able to simplify them enough without oversimplifying them. This can lead experts to appear obscure or distant to the general public. But because pseudoexperts are not bound by their respect for truth, or indeed simply do not know the truth, they might be less hesitant to oversimplify, which will allow them to induce positive feelings of clarity, fluency and understanding in their public (Nguyen, 2021). For example, Tetlock (2005) and Tetlock and Gardner (2016) argues that “hedgehog experts” who stick to a handful of Big Ideas that allow them to explain everything are more likely to be favored by the media, even if their predictions tend to be worse. This is because “hedgehogs tell tight, simple, clear stories that grab, and hold audiences” (Tetlock and Gardner, 2016).

#### Meeting the Attractiveness Challenge: Conflict of Interests and Other Brands of Moral Grandstanding

When assessing the value of a given testimony, skills and competence are not the only things we take into account – we also evaluate how trustworthy our interlocutor should be considered. In the case of experts, questions of trustworthiness raise concern about experts’ conflicts of interest: do experts have any personal interest in deceiving us? This is a legitimate worry (as highlighted above by Goldman’s criteria D), but a worry that might be used by pseudoexperts to discredit other experts and get the upper hand (on the validity of such arguments, see Zenker, 2011). Most pseudoexperts can easily stress their independence, as most of them do not belong to official research institutions anyway or are not servants of governmental or private interests (as neither the government nor enterprises have any reason to fund them). Moreover, stressing other experts’ conflict of interests might be a way to defuse the objections these other, genuine experts might have raised against the pseudoexpert’s claims. For example, in the context of the debate about hydroxychloroquine, Didier Raoult frequently mentioned his opponents’ financial ties to pharmaceutical companies. He went as far as publishing a manuscript suggesting an implausibly strong correlation (*r* = 1.00) between physicians’ conflicts of interest with the pharmaceutical company Gilead Sciences and their opposition to hydroxychloroquine as a treatment against COVID-19 (Roussel and Raoult, 2020). And in the case pseudoexperts who are members of scientific institutions are criticized by their colleagues, they can style themselves as mavericks or whistle-blowers (Brock, 1968; Fromkin, 1972).

In fact, morality might appear to non-experts as a less opaque quality than competence, skills and credentials. As Goodwin (2010) puts it, “the skill of judging trustworthiness is widely distributed; indeed it’s available to anyone who is willing to devote some time to practicing it in their everyday life.” Thus, we can expect pseudoexperts to try and distinguish themselves from their competitors by their morality. This can be done by ostensibly engaging in the defense of moral causes or by grandstanding (Tosi and Warmke, 2016).

Thus, we can expect pseudoexperts to emphasize their own morality and the immorality of genuine experts, in particular when it comes to conflicts of interest.

### What Is the Typical Dynamics of Pseudoexpertise?

A final line of research might investigate what becomes of pseudoexperts in the long run and how stable a phenomenon like pseudoexpertise can be. Here, we would like to argue for the hypothesis that pseudoexpertise is a volatile phenomenon and that pseudoexperts tend to degenerate in other kinds of obstacles to knowledge. By its very nature, pseudoexpertise tends to be short-lived.

Indeed, since the typical pseudoexpert seeks the exposure that is necessary to reap the benefits that come with the status of experts, it is highly likely that, sooner or later, they will be confronted by genuine experts. What happens next will depend on the pseudoexpert’s situation at the moment the criticisms begin to appear. If the pseudoexpert’s status is by that time already well-established, they might not need to do anything at all, except maybe shrug criticisms off, for example by saying that these are only spiteful attacks from jealous people. As long as they keep on managing to be recognized as experts by their audience, they do not need to confront people who enjoy no such recognition, or a much lesser one, among non-experts.

Still, it is sometimes impossible to simply ignore or silence criticisms, especially in the age of social media. The fate of pseudoexpertise in such cases is yet an interesting matter for further study. Following proposals made in the context of pseudoscientific rhetoric and behavior, we might speculate that pseudoexperts will resort to strategies of epistemic immunization, and double-down on the aforementioned strategies of legitimation. To salvage and reinforce their appearance of competence and trustworthiness, they might resort to further use of ambiguous concepts, multiply the number of their predictions and pronouncements, construct *ad hoc* hypotheses, generate internal explanations for the reasons of the attacks they undergo, or simply uptake a conspiratorial logic (Boudry and Braeckman, 2011; Talmont-Kaminski, 2013). At worse, this attitude would lead to a polarization of the initial claims and posture, and at this point the pseudoexpert would turn into something else entirely: perhaps a guru if there is a pronounced enough cultic and conspiratorial drift. Yet, in most cases, we suspect that most situations of pseudoexpertise will simply go away: the pseudoexpert has been unmasked, the window of opportunity has waned, other pseudoexperts have redirected the public’s attention, and so on. The dynamics of pseudoexpertise, it seems to us, are currently poorly understood: it might be that the most paradigmatic and visible cases are the exception, and pseudoexpertise is usually very short-lived, simply because genuine expertise is valued and needed in our societies.

## Conclusion

In this manuscript, we have attempted to delineate the contours of a ubiquitous, yet poorly researched, figure of public life: the pseudoexpert. We have tried to identify this phenomenon by providing a working definition derived from a conceptual analysis of previous uses of the term “pseudoexpertise” and based on recent philosophical work on the concept of expertise. Although pseudoexpertise can be easily mistaken for pseudoscience, incompetence or mere overconfidence, we have argued that pseudoexpertise should be distinguished from these adjacent phenomena, and that real cases of pseudoexpertise, although very diverse, are best understood under our characterization.

Our method was based on conceptual analysis starting from well elaborated philosophical approaches to expertise – an ability to serve a specific function in the division of epistemic labor that is grounded in knowledge and skills in a specific domain and accompanied by social recognition. Criteria for expertise are selectively mimicked by the pseudoexpert, in an attempt to pass as an expert in the eyes of the general public, and thus abuse its trust in order to dispense unreliable information and reap personal benefits (be they material, social, or psychological).

As such, we think that the study of pseudoexpertise might help better understand the nature of genuine expertise. For instance, we have argued that a purely reputational approach to expertise, in that experts are merely people recognized as experts by the public and nothing else, would be unable to account for the existence of pseudoexperts. On such a narrow view of expertise, pseudoexperts would merely be people pretending to be *recognized* as experts, which seems unsatisfactory. Rather, on our account, pseudoexperts *are* recognized, though unduly, as experts, and that explains why pseudoexpertise might be attractive to some people, and also a problem to society. More generally, understanding the strategies used by pseudoexperts, and the challenges they have to meet in order to successfully pass as experts, should help us better delineate the building blocks of genuine expertise, both as a capacity involving competence and skills, and a social function involving trust and reputation.

In a second step, we have presented a framework for further research based on three questions. If we are right that there exists a phenomenon of pseudoexpertise, such as we have defined it, then we should try to understand how it exists at all, how it works and how it evolves in time. To do so, we have highlighted several strands of existing research and theory broadly derived from the field of cultural cognition and evolution. Future research could exploit this framework to investigate how the general public can sometimes be misled by information they attribute to experts, when in fact it has been produced by pseudoexperts. The very fact that pseudoexpertise can sometimes be successful raises interesting avenues for research on the perception of competence, on the mechanisms of reputation and on the features of expert-like discourse.

Pseudoexpertise is a problem for open and liberal societies, in which people must rely on expert knowledge and skills, and thus on the trust we afford to those we see as genuine experts. Much of the expert responses we crucially need during severe crises, such as the COVID-19 pandemic, will be influenced and slowed-down by the intrusion of claims and advice presenting themselves as expertise, but in fact only pretending to be so. How pseudoexperts can be taken seriously, and how long their pretense can last, are questions that urgently need to be addressed. Here we have provided a first attempt to better understand the problem of pseudoexpertise.

## Data Availability Statement

The original contributions presented in the study are included in the article/supplementary material, further inquiries can be directed to the corresponding author.

## Author Contributions

All authors listed have made a substantial, direct and intellectual contribution to the work, and approved it for publication.

## Conflict of Interest

The authors declare that the research was conducted in the absence of any commercial or financial relationships that could be construed as a potential conflict of interest.

## Publisher’s Note

All claims expressed in this article are solely those of the authors and do not necessarily represent those of their affiliated organizations, or those of the publisher, the editors and the reviewers. Any product that may be evaluated in this article, or claim that may be made by its manufacturer, is not guaranteed or endorsed by the publisher.

## Footnotes

1 See 4ème oeil corporation, https://www.youtube.com/channel/UCu4qNTXsi60RooRszZZUD-g/videos.

2 Collins (2018) hints at a third type of expertise. Indeed, Collins distinguishes between “contributory” and “relational” expertise. “Contributory expertise” is defined as “what we normally mean when we talk of experts—these are people who exercise their expertise by contributing to their specialist domain” (Collins, 2018, p. 72), and thus can be identified with Croce’s *research-oriented* approach to experts. “Relational expertise” is defined as the expertise acquired by people who have interacted with the field enough to gain a knowledge and a mastery of the technical language similar to those of pseudoexperts, without contributing to the field themselves. Collins offers himself as an example: as a sociologist of science, he interacted with specialists in gravitational waves long enough to gain a deep knowledge of the field, without directly contributing to it. “Relational experts” in this sense include cases of *novice-oriented* experts that are not *research-oriented* experts, such as science popularizers and journalists (to the extent that they have real knowledge of their topic). However, for Collins, “relational experts” extend beyond such cases to cover cases of “peer review where the reviewer will rarely have practiced the practices whose worth is being judged” and “committee meetings judging technical issues” (p. 73). We think that an appropriate definition of pseudoexperts should take care not to dismiss such cases as pseudoexperts.

3 Whether science journalists count as “experts” in their domain will depend on where we set the competence threshold for one to count as an expert. On a purely comparative account of expertise, we could say that science journalists are experts compared to laypeople but “less” experts than scientific researchers that are directly involved in the latest advances of their field.

4 One might of course suggest more complex taxonomies of pseudoexpertise. For our purposes here, this simple dichotomy will suffice, but we take pseudoexpertise to be a very flexible, dynamic and versatile phenomenon, and so a careful examination of specific case studies, perhaps in different cultural contexts, might very well end up with many faces of pseudoexpertise, relying on multiple mechanisms and influences.

5 Note that, on our definition, pseudoexperts need not make mistakes of make false claims: we could imagine a *careful* pseudoexpert who, once settled, is content with making very trivial claims about their field, while avoiding bold, dubious statements. However, we argue in section “What Strategies Make Pseudoexperts Successful?” that the pressure of being attractive might motivate pseudoexperts to make cognitively attractive statements that are bound to be exaggerated or provocative.

6 Some of these suggestions overlap with, but are not identical to the strategies used by “denialists” and identified by Diethelm and McKee (2009).

7 The Evolution of Confusion https://www.youtube.com/watch?v=D_9w8JougLQ&ab_channel=RichardDawkinsFoundationforReason%26Science.
